# Supplementary material for: Myosin II activity is required for structural plasticity at the axon initial segment
Source: Eur J Neurosci. 2017 May 17;46(2):1751–7. doi: 10.1111/ejn.13597 (PMC5573965; doi:10.1111/ejn.13597)
Supplement: Supplementary file 1 [file EJN-46-1751-s001.pdf]

EJN European Journal of Neuroscience FENS Federation of European Neuroscience Societies

Myosin II activity is required for structural plasticity at the axon initial segment

Mark D Evans, Candida Tufo, Adna S Dumitrescu & Matthew S Grubb

Review timeline:

Submission date:	19 January 2017
Editorial Decision:	15 February 2017
Revision received:	29 March 2017
Accepted:	20 April 2017

Editor: Patricia Gaspar

1st Editorial Decision 15 February 2017

Dear Matt,

Your manuscript was reviewed by four (!) external reviewers as well as by the Section Editor, Dr. Patricia Gaspar, and ourselves. We are pleased to say that the reviews collectively indicate that your experiments generated new and important information. However, there are several issues that need to be clarified/resolved before we can consider your manuscript further for publication in EJN.

All 4 reviewers recommended your work as containing important original contribution that is worthy of publication. They however made several remarks that need to be addressed. Most remarks relate to the interpretation of the data , such as the fact that the experiments do not allow to state that myosin is not downstream of calcineurin signaling. However additional data such as a demonstration of the localisation of myosinII at the AIS, would be more convincing to support the notion of a direct effect.

If you are able to respond fully to the points raised, we would be pleased to receive a revision of your paper within 30 days.

Thank you for submitting your work to EJN.

Kind regards,

Paul & John
co-Editors in Chief, EJN

Reviews:

Reviewer: 1 (Christine Metin, CNRS UMR 8542, Equipe Regionalisation Nerveuse)

Comments to the Author
The laboratory of Matthew Grubb previously demonstrated that long-term neuronal depolarization produces a distal shift of the AIS away from the cell body, and that short-term depolarization produces a shortening of the AIS. The distal shift associates with a decrease in neuronal excitability. AIS changes involve the activation of voltage-gated calcium channels and downstream signaling events mediated by the calcium-sensitive phosphatase calcineurin.

EJN European Journal of Neuroscience FENS Federation of European Neuroscience Societies

In the present study, Grubb and collaborators used blebbistatin, a specific inhibitor of myosin 2, to show that AIS shift or shortening induced by depolarization requires the activity of myosin 2. They show that calcineurin activation is not perturbed by myosin 2 inhibition and that AIS shortening does not depend on endocytosis. They conclude from these results that myosin II is required for AIS plasticity and discuss several putative mechanisms.
Authors are using an experimental model (cultures of dissociated cells form the dentate gyrus) that they perfectly master, for a long time. Data are analyzed in a very rigorous way, and figures are very clear with beautiful illustrations. The paper is very well written.
I have however, two main concerns:
- Figure 4 illustrates a negative result showing that myosin II does not regulate AIS plasticity by controlling endocytosis. They are several other hypotheses, and I am not sure that studies invalidating a wrong hypothesis deserve a whole figure.
- In my opinion, the main weakness of the paper is that neither myosin 2 nor MLCK, the regulatory kinase of myosin 2, have been localized in these cultured neurons. Are they present within the AIS, in the PAEZ, far away from the AIS, ...? How do they distribute in blebbistatin- and KCl-treated neurons? Is F-actin distribution affected in these experimental conditions? These informations are mandatory to support a functional role of myosin 2 in regulating AIS plasticity, and to further elaborate mechanistic hypotheses. Antibodies against Myosin 2 and against the phospho-MLCK are available.

Reviewer: 2 (Christophe Leterrier, Aix-Marseille Universite, NICN CNRS UMR 7259)

Comments to the Author
This short communication by Evans et al. is straightforward: the AIS morphological plasticity is blocked by inhibition of myosin II activity with blebbistatin. Both long-term shift away from the soma and short-term shortening are affected. A few mechanistic insights are also provided: myosin action happens downstream of calcineurin, and does not involve endocytosis.

I think this is a very interesting finding, and like all interesting results, it brings more questions than answers (to be clear I think it is a good thing for a short report). One can hope that follow-up studies will explore the mechanistic and structural implications of this finding.

I have only minor comments that I would like to submit to the authors before recommending publication in the European Journal of Neuroscience.

• The distance between the soma and the calculated starting point of the AIS is an important measurement for the 48h plasticity. However it is not clear how the starting point of the axon is determined with the reported staining for Px1 and ankG. For example on the Figure 1 top left panel, the axon starts (thick bar) somewhere between the Px1 somatic labeling and the AIS ankG labeling, but how is this chosen when tracing the axon?
• Blebbistatin is known to be toxic. Could the author precise if signs of neuronal toxicity were present after 48h in the presence of 50 µM blebbistatin, and/or cite other studies that have used similar long-term treatments?
• In the results, the author state that there is a significant difference between DMSO and blebbistatin in the presence of KCl for NFAT-GFP distribution (“Tukey post-test for DMSO vs blebbistatin in KCl, $p<0.0001$”, which is the interesting point), but the graph only shows significance stars for NaCl vs KCl in DMSO and blebbistatin conditions. Would it be possible to add that difference on the graph?
• In the discussion, I’m not sure the main hypothesis of a myosin action on antiparallel bundles within hotspots and patches is the most plausible. There are few hotspots in the AIS, if as in Ganguly et al. JCB 2015 you call hotspots the actin clusters that nucleate trails. Moreover, the nature of patches seen in live cells is still debated. They may well correspond to GABA postsynaptic specializations (d’Este et al. Cell Rep 2015), and these postsynaptic sites don’t move when the AIS shifts distally in plasticity experiments (Muir

Front Cell Neuro 2014, Wefelmeyer PNAS 2015). So maybe the current “alternative” hypothesis, action of myosin at the level of submembrane rings where actin couples to the ankG scaffold via ß4-spectrin would be the most plausible mechanism?

Reviewer: 3 (Pirta Hotulainen, Minerva Foundation Institute for Medical Research)

Comments to the Author
Evans et al. describes a novel role for myosin II in AIS plasticity. It is nowadays well known that AIS undergoes neuronal activity induced plastic changes in its length and location. However, it is not at all clear how these changes can be achieved. The calcineurin activation seems to be important, but what is occurring down-stream of calcineurin or through other pathways/mechanisms, is not known. Therefore, I find the discovery presented in this manuscript important. This is the first time to show the involvement of a protein in AIS plasticity, which can through its own action change the structure. Furthermore, the discovery suggests that regulation of the actin structures is involved in controlling the AIS plasticity. Manuscript presents the main finding without going to deeper mechanistic analyses. I can think a long list of “next experiments” but obviously authors wanted to publish this result as such. And this result as such is valuable and I am sure that scientists focusing more on actin regulation in AIS are happy to continue from this point. Manuscript is nicely written, experiment are carefully carried out and discussion goes through the main aspects.
I have only one correction suggestion. In the end of the third paragraph in results, authors conclude results: “Myosin II is therefore required for structural AIS plasticity at some point downstream of calcium dependent calcineurin signaling.” This sounds like myosin II is activated by calcineurin, but the results only show that myosin II activity do not affect activation of calcineurin. Therefore it is good to rephrase this sentence to avoid confusion.

Reviewer: 4 (Xavier Nichol, Institut de la Vision, Inserm-UMR-S 968)

Comments to the Author
The study of Evans et al is a follow-up of previous reports from the same group focusing on activity-dependent structural plasticity at the axon initial segment (AIS). The authors investigate the cellular mechanisms involved, focusing on myosin II. Using exclusively the myosin II ATPase inhibitor blebbistatin, they demonstrate that myosin II activity is required for structural plasticity.
Overall the manuscript is clear, concise and well illustrated. Most of the claims are supported by the data. The lack of clear data demonstrating a direct interaction between Myosin II and structural plasticity is slightly disappointing. Myosin II might just be a component of a motility-regulating pathway that is permissive to but does not regulate AIS-dependent structural plasticity per se (see point #2).

Major points:
#1. The authors should provide localization data describing the subcellular distribution of Myosin II compared to an AIS marker. An enrichment of Myosin II at the AIS would strengthen their finding that are for now exclusively based on a single pharmacological agent.

#2. The title of the manuscript reflects the data provided, stating that Myosin II is required for structural plasticity. However in several occurrence in the text, the authors claim stronger conclusion, suggesting that Myosin is part of a signaling pathway controlling structural plasticity (e.g. page 8 line 5 “How might Myosin II be acting to produce activity-dependent structural changes at the AIS?”). Myosin activity might alternatively be involved in a cellular function that is permissive for AIS plasticity. For instance, Myosin might provide a basal motility to the cytosqueleton. If blocked (by blebbistatin), the lack of motility would prevent any change in the AIS length or position, altering structural plasticity through a permissive mechanism rather than involving a direct control of AIS. This possibility is shortly acknowledge in the discussion but quickly eliminated based on a weak “parsimony” explanation.

EJN European Journal of Neuroscience FENS Federation of European Neuroscience Societies

#3. Based on the absence of NFAT-GFP nuclear relocalization defect when blebbistatin is applied, the authors claim that “Myosin II is required for structural AIS plasticity at some point downstream of calcium-dependent calcineurin signaling”. The conclusion should rather be that Myosin II is not involved upstream of calcineurin. It can still be required in a permissive pathway, or in pathway yet unidentified regulating structural plasticity in parallel to calcineurin (see comment #2).

Minor points.
#4. The quantification method used to evaluate the length of the AIS is subject to caution. The extremities of the AIS are determined as the position where the fluorescence intensity is under 33% of the maximum fluorescence in the axon (using the Ankyrin G staining). The threshold used (33%) is likely not to impact much the position of the proximal end of the AIS since the decrease of fluorescence is sharp. In contrast, the distal end of the AIS is less clearly marked. Would the effect of blebbistatin be affected by a change in this threshold value (Figure 2)?

#5. In Figure 2, the Y scale of the graph plotting the average AIS length does not start from 0. This misrepresent the amplitude of the detected variation in AIS length. A Y scale starting from 0 should be provided.

Authors' Response 29 March 2017

Responses to Reviewers
Dear Patricia, Paul and John

We thank all four Reviewers for their positive and constructive comments on our manuscript. In response, we have generated new data and a new figure showing myosin II localisation in our neurons. We have also thoroughly re-analysed our data to discount any effects of our chosen AIS quantification threshold, and have re-written various parts of the manuscript to address concerns in detail and interpretation. We believe that we have now fully addressed the issues raised by all Reviewers, and hope that you and they consider our manuscript suitable for publication in EJN in its new form.

A point-by-point response to each Reviewer is enclosed below. We look forward to hearing back from you soon

Reviewer 1
We are pleased that this Reviewer thought our ‘*Data are analyzed in a very rigorous way, and figures are very clear with beautiful illustrations. The paper is very well written.*’ They had two concerns:

1) *Figure 4 illustrates a negative result showing that myosin II does not regulate AIS plasticity by controlling endocytosis. They are several other hypotheses, and I am not sure that studies invalidating a wrong hypothesis deserve a whole figure.*

We appreciate that Figure 4 presents a negative finding, but we do believe it to be an important one. Bulk endocytosis is one documented example of an activity-dependent neuronal process that requires both calcineurin signalling and myosin II activity, just like structural AIS plasticity. It is also relatively easy to image a scenario in which bulk endocytosis could be involved in structural AIS plasticity, especially in the case of rapid AIS shortening. We therefore believe the finding that dynasore does not block activity-dependent AIS shortening deserves its place in our final figure.

2) In my opinion, the main weakness of the paper is that neither myosin 2 nor MLCK, the regulatory kinase of myosin 2, have been localized in these cultured neurons. Are they present within the AIS, in the PAEZ, far away from the AIS, ….? How do they distribute in blebbistatin- and KCl-treated neurons? Is F-

actin distribution affected in these experimental conditions? These informations are mandatory to support a functional role of myosin 2 in regulating AIS plasticity, and to further elaborate mechanistic hypotheses. Antibodies against Myosin 2 and against the phospho-MLCK are available.

We now present immunocytochemical data in a new Fig. 2 (along with accompanying additional text in the Methods, Results and Discussion) that demonstrate the distribution of myosin IIb in our cultured neurons. We find that myosin IIb is not specifically localised to or concentrated at the AIS, but that it is present at the structure in all of our experimental conditions. This at least allows for the possibility that myosin II's involvement in AIS plasticity might occur through local action at the structure itself.

Reviewer 2

This Reviewer was also very positive: '*I think this is a very interesting finding, and like all interesting results, it brings more questions than answers (to be clear I think it is a good thing for a short report). One can hope that follow-up studies will explore the mechanistic and structural implications of this finding.*' They had several minor comments:

1) *The distance between the soma and the calculated starting point of the AIS is an important measurement for the 48h plasticity. However it is not clear how the starting point of the axon is determined with the reported staining for Px1 and ankG. For example on the Figure 1 top left panel, the axon starts (thick bar) somewhere between the Px1 somatic labeling and the AIS ankG labeling, but how is this chosen when tracing the axon?*

The point where the axon emanates from the soma is actually sufficiently obvious from background label in the AnkG channel (especially when the brightness in this channel is enhanced during the axon tracing process). This is now detailed in the Methods section (p4).

2) *Blebbistatin is known to be toxic. Could the author precise if signs of neuronal toxicity were present after 48h in the presence of 50 µM blebbistatin, and/or cite other studies that have used similar long-term treatments?*

We saw no signs of neuronal toxicity, even after 48 h in 50 µM blebbistatin. This is in keeping with other studies using similar concentrations of the drug over similar time periods in dissociated hippocampal cultures – for example the Kollins et al. (2009) paper cited in our original manuscript, where chronic myosin II inhibition actually enhanced neurite growth in young neurons. We can also be reasonably sure that our neurons were in good condition because, as well as them showing no overt signs of ill-health, the structural integrity of the AIS is actually an excellent indicator of neuronal health – AISs rapidly degrade when cells are unhealthy (Schafer et al. 2009, now cited in our revised manuscript), and we saw no effects of blebbistatin on our AISs under baseline conditions. This is all now detailed in the Methods section (p3).

3) *In the results, the author state that there is a significant difference between DMSO and blebbistatin in the presence of KCl for NFAT-GFP distribution ("Tukey post-test for DMSO vs blebbistatin in KCl, p<0.0001", which is the interesting point), but the graph only shows significance stars for NaCl vs KCl in DMSO and blebbistatin conditions. Would it be possible to add that difference on the graph?*

This comparison has now been added to the graph as requested.

4) *In the discussion, I'm not sure the main hypothesis of a myosin action on antiparallel bundles within hotspots and patches is the most plausible. There are few hotspots in the AIS, if as in Ganguly et al. JCB 2015 you call hotspots the actin clusters that nucleate trails. Moreover, the nature of patches seen in live cells is still debated. They may well correspond to GABA postsynaptic specializations (d'Este et al. Cell Rep 2015), and these postsynaptic sites don't move when the AIS shifts distally in plasticity experiments (Muir*

Front Cell Neuro 2014, Wefelmeyer PNAS 2015). So maybe the current "alternative" hypothesis, action of myosin atthe level of submembrane rings where actin couples to the ankG scaffold via ß4-spectrin would be the most plausible mechanism?

We have now re-worked this paragraph of the Discussion (p8-9) so that the ring-based possibility is presented first, but also to make it as clear as possible that either or both potential actin-based mechanisms are currently equally plausible.

Reviewer 3

This Reviewer finds '*the discovery presented in this manuscript important.*' They are also of the opinion that the '*Manuscript is nicely written, experiment are carefully carried out and discussion goes through the main aspects.*' They have one suggestion:

1) In the end of the third paragraph in results, authors conclude results: "Myosin II is therefore required for structural AIS plasticity at some point downstream of calcium dependent calcineurin signaling." This sounds like myosin II is activated by calcineurin, but the results only show that myosin II activity do not affect activation of calcineurin. Therefore it is good to rephrase this sentence to avoid confusion.

We have rephrased this sentence (p7) and hope it is now much clearer.

Reviewer 4

We are pleased that this Reviewer found the manuscript to be '*clear, concise and well illustrated.*'

They made the following general comment:

The lack of clear data demonstrating a direct interaction between Myosin II and structural plasticity is slightly disappointing. Myosin II might just be a component of a motility- regulating pathway that is permissive to but does not regulate AIS-dependent structural plasticity per se (see point #2).

We agree entirely that, whilst demonstrating an essential role for myosin II in AIS plasticity, the current manuscript does not provide any data regarding the mechanism(s) by which this occurs. We have tried to be extremely careful throughout the paper to never pretend otherwise, and have made changes to the revised manuscript to ensure this (see below). However, especially for a Short Communication paper, we believe that the basic finding of myosin II's crucial involvement in these phenomena is of sufficient interest to warrant publication in and of itself. We believe we are supported in this opinion by the other Reviewers and the Editors.

Major points:

1) The authors should provide localization data describing the subcellular distribution of Myosin II compared to an AIS marker. An enrichment of Myosin II at the AIS would strengthen their finding that are for now exclusively based on a single pharmacological agent.

We have now provided these data in the new Fig. 2 (see also our response to Reviewer 1, #2 above). We do not find an enrichment of myosin II at the AIS, but we do find puncta that are co -localised with AnkG. These new data do not strengthen our main finding, or definitively point to a mechanism of myosin II action, and we try to avoid any such claims in the manuscript. They do, however, lend some plausibility to the idea that myosin II's role in AIS plasticity might, at least in part, be a local one.

EJN European Journal of Neuroscience FENS Federation of European Neuroscience Societies

2) *The title of the manuscript reflects the data provided, stating that Myosin II is required for structural plasticity. However in several occurrence in the text, the authors claim stronger conclusion, suggesting that Myosin is part of a signaling pathway controlling structural plasticity (e.g. page 8 line 5 "How might Myosin II be acting to produce activity-dependent structural changes at the AIS?"). Myosin activity might alternatively be involved in a cellular function that is permissive for AIS plasticity. For instance, Myosin might provide a basal motility to the cytosqueleton. If blocked (by blebbistatin), the lack of motility would prevent any change in the AIS length or position, altering structural plasticity through a permissive mechanism rather than involving a direct control of AIS. This possibility is shortly acknowledge in the discussion but quickly eliminated based on a weak "parsimony" explanation.*

We have now re-written the highlighted section of the Discussion (p8), and have paid great attention to our language throughout the text to avoid any suggestion that our data point to any particular mechanism of action.

3) *Based on the absence of NFAT-GFP nuclear relocalization defect when blebbistatin is applied, the authors claim that "Myosin II is required for structural AIS plasticity at some point downstream of calcium-dependent calcineurin signaling". The conclusion should rather be that Myosin II is not involved upstream of calcineurin. It can still be required in a permissive pathway, or in pathway yet unidentified regulating structural plasticity in parallel to calcineurin (see comment #2).*

We agree entirely and have altered the relevant sentence accordingly (p7).

Minor points:

4) *The quantification method used to evaluate the length of the AIS is subject to caution. The extremities of the AIS are determined as the position where the fluorescence intensity is under 33% of the maximum fluorescence in the axon (using the Ankyrin G staining). The threshold used (33%) is likely not to impact much the position of the proximal end of the AIS since the decrease of fluorescence is sharp. In contrast, the distal end of the AIS is less clearly marked. Would the effect of blebbistatin be affected by a change in this threshold value (Figure 2)?*

We re-analysed all of our blebbistatin experiment data using a range of threshold values (0.1, 0.2, 0.3, 0.4, 0.5), and in every case found an identical result in terms of significance for every statistical test reported in the manuscript. This is now explained briefly in the Methods section (p4). We also present these data and analyses for the Reviewer in the figure and table below:

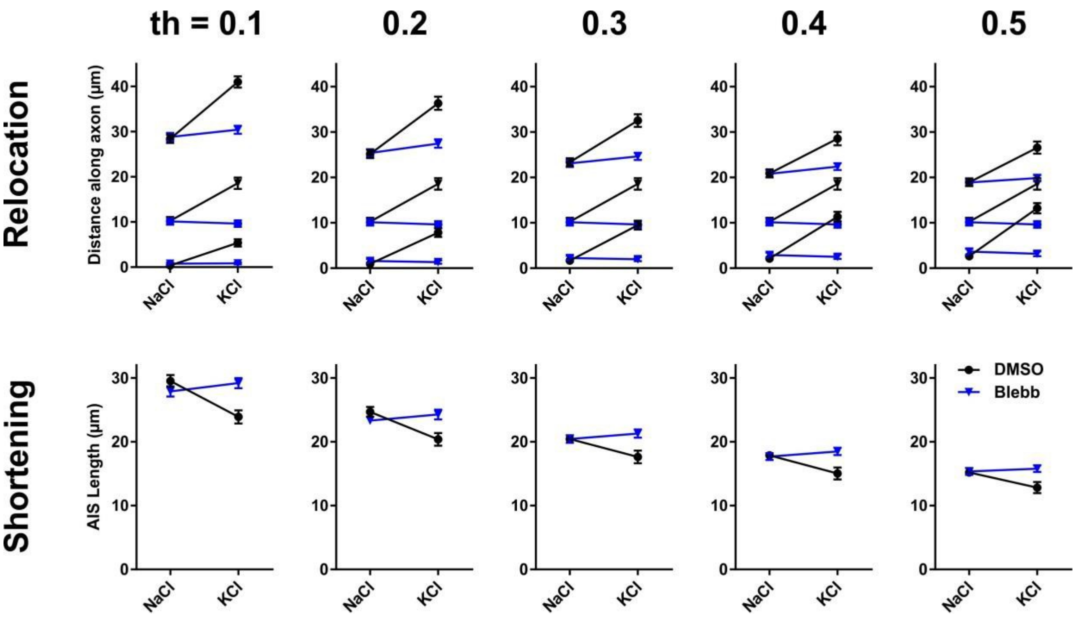

Figure R1. Blebbistatin blocks AIS plasticity as measured over a range of AIS thresholds.

Threshold	AIS Relocation (Tukey after 2-way ANOVA)								AIS shortening (Tukey after 2-way ANOVA)	
	Start		Max		End		AMI			
	DMSO	Blebb	DMSO	Blebb	DMSO	Blebb	vs 1	vs 0	DMSO	Blebb
**0.1**	<0.0001	0.82	<0.0001	0.99	<0.0001	0.44	<0.0001	0.48	<0.0001	0.65
**0.2**	<0.0001	0.97	<0.0001	0.99	<0.0001	0.33	<0.0001	0.48	0.0008	0.82
**0.3**	<0.0001	0.99	<0.0001	0.99	<0.0001	0.40	<0.0001	0.48	0.026	0.83
**0.4**	<0.0001	0.99	<0.0001	0.99	<0.0001	0.33	<0.0001	0.48	0.0094	0.83
**0.5**	<0.0001	0.99	<0.0001	0.99	<0.0001	0.62	<0.0001	0.48	0.020	0.97
**0.33**	<0.0001	0.99	<0.0001	0.99	<0.0001	0.58	<0.0001	0.59	0.0054	0.84

Table R1. P-values from post-hoc statistical tests of blebbistatin experiments with AISs measured over a range of AIS thresholds.

5) *In Figure 2, the Y scale of the graph plotting the average AIS length does not start from 0. This misrepresent the amplitude of the detected variation in AIS length. A Y scale starting from 0 should be provided.*

The Reviewer refers to the inset mean+-SEM graph in this figure (now Figure 1B), where the main data plot showing the full distribution of all datasets does have an axis starting from zero. The main point here is not to overstate the degree of AIS shortening – again, this is shown in full detail in the main cumulative distribution plot – but to highlight the complete lack of shortening in the presence of blebbistatin. We respectfully disagree that this would be made any clearer or more accurate by expanding the y-scale of the inset plot, and have left the graph unchanged.
